# Low-Cost Servomotor Driver for PFM Control

**DOI:** 10.3390/s18010093

**Published:** 2017-12-31

**Authors:** David Aragon-Jurado, Arturo Morgado-Estevez, Fernando Perez-Peña

**Affiliations:** School of Engineering, University of Cadiz, PC 11519 Puerto Real, Cadiz, Spain; david.aragon@uca.es (D.A.-J.); arturo.morgado@uca.es (A.M.-E.)

**Keywords:** PFM, servomotor, robotics, neurorobotics, potentiometer

## Abstract

Servomotors have already been around for some decades and they are extremely popular among roboticists due to their simple control technique, reliability and low-cost. They are usually controlled by using Pulse Width Modulation (PWM) and this paper aims to keep the idea of simplicity and low-cost, while introducing a new control technique: Pulse Frequency Modulation (PFM). The objective of this paper is to focus on our development of a low-cost servomotor controller which will allow the research community to use them with PFM. A low-cost commercial servomotor is used as the base system for the development: a small PCB that fits inside the case and allocates all the electronic components to control the motor has been designed to replace the original. The potentiometer is retained as the feedback sensor and a microcontroller is responsible for controlling the position of the motor. The paper compares the performance of a PWM and a PFM controlled servomotor. The comparison shows that the servomotor with our controller achieves a faster mechanism for switching targets and a lower latency. This controller can be used with neuromorphic systems to remove the conversion from events to PWM.

## 1. Introduction

Servomotors are electromechanical devices manufactured with a DC motor, a gear set, a control circuit, a potentiometer and a plastic case that covers the system [1]. The main characteristic of these devices is their capacity to move to a set position (measured in degrees) due to the feedback produced by the internal potentiometer.

Servomotors were proposed by Calendar in 1896 [2] in England, who developed the first electric servo-mechanism device. Due to their easy control method, they are extremely popular among roboticists, who use their capabilities for different types of projects, e.g., the robotic goalie arm [3], or the Honda humanoid robot [4].

There are plenty of studies about humanoid robot development where servomotors are used [5,6]. In addition, there are studies where servomotors are used for other projects, such as a tactile shape display [7].

Currently, most servomotors are controlled using Pulse Width Modulation (PWM) [8]. This modulation encodes the message (in this case, the amount of degrees) producing a square signal where there is a relation between the message and the duty-cycle (the amount of time where the signal is active). This signal has a fixed frequency and a configurable duty-cycle, which enables modification of the shaft position of the servomotor.

**Figure 1:**
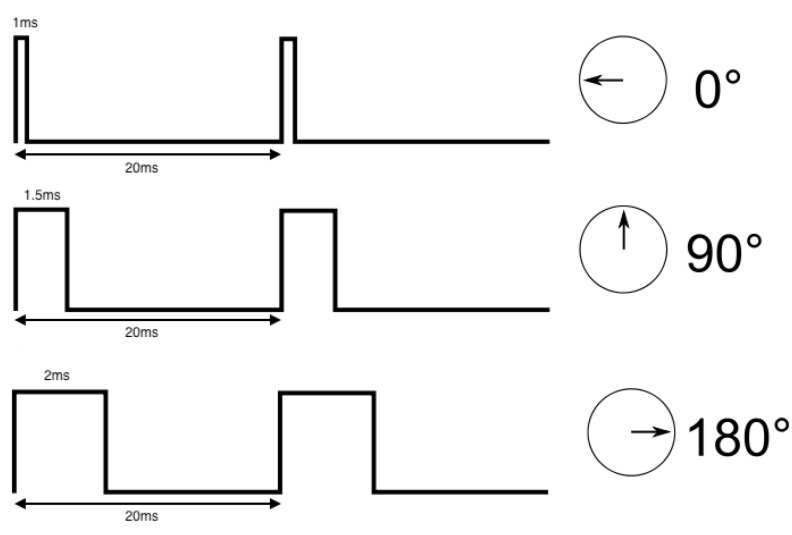
Example of the relation between the duty-cycle of the square control signal and the degree of rotation of a Pulse Width Modulation (PWM) servomotor.

The control circuit of a servomotor sets the position that the shaft has to reach. There is a relation between the duty-cycle of the square signal and the desired rotation of the servomotor. The control circuit uses that relation. Each device has its operation limits, which correspond to the maximum and minimum duty-cycle that the servomotor controller can generate. For the example shown in Figure 1, the PWM signal to control the motor is within the range of 1–2 ms width pulses, or 5–10% of duty-cycle, which corresponds to 0–180°, the most common range used by servomotor controllers [9].

Figure 1 shows an example of the operation of a servomotor. For this example, the frequency of the PWM signal is 50 Hz and the full operation range goes from a duty-cycle of 5% to 10% (a pulse-width going from 1 to 2 ms) of the signal, meaning a rotation range from 0° to 180° (the limit tab will set the degrees rotation range). As shown, the shaft will turn to its center position when the controller generates a squared signal with a duty-cycle of 7.5% (equivalent to a pulse width of 1.5 ms).

Nowadays, the bioinspired research field is growing quickly. This field aims to imitate the behavior and performance of any biological system to use their features for a predetermined purpose [10,11,12,13]. For that, they try to emulate different behaviors or dynamics of different biological systems. Some previous works involving low-level motor control used PWM. They used a microcontroller to read the output of the neural population and then converted the rate of the neurons into the PWM message. Thus, this method uses an extra device as well as introduces delays to the system [14].

The objective of this paper is to design and implement a new controller for servomotors. The novelty of this paper is to provide a driver to control the servomotors using Pulse Frequency Modulation (PFM). This modulation is a control system normally used as a control method of DC motors, modulating their speed or position as a function of the frequency of the signal.

The reason for taking this approach is that PFM is becoming popular for several fields: bioinspired systems, neurorobotics and neruromorphic engineering, among others [15,16]. With our driver, the firing rate of a neuron model could be directly used to drive the motor, removing the problem of using extra devices.

Studies can be found where PFM is used as a signal of feedback in a control system, as Bernard Friedland explains [17]. Reports where PFM is used as control of the voltage frequency of inverters are also found [18].

Works that are closer to our aim are the ones using PFM: bioinspired systems, such as retinomorphic sensors [19], prosthetic feedback [20], neuro-inspired controller [21] or spike-based PID controllers [14].

To our knowledge, there are no previous works or reports on servomotors controlled using PFM.

**Figure 2:**
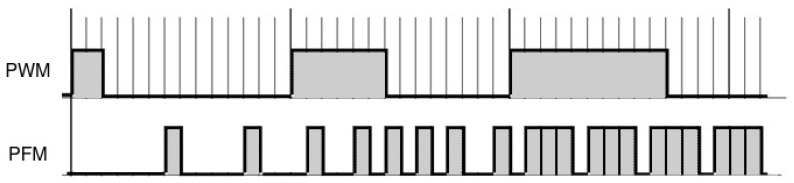
Comparison between PWM and Pulse Frequency Modulation (PFM) signals.

Unlike PWM control, when using PFM, the duty-cycle remains constant and the frequency is variable, as shown in Figure 2. The information is encoded within the frequency of the square signal, in a similar way to what can be found in some biological nervous units, such as the spindles, motorneurons, etc. [22].

This project arises from the idea of creating a bioinspired servomotor. For that, the starting point of the study is the control method of the servomotor, which is usually PWM control. We aim to replace this method of control with PFM control, which is a method commonly used in bioinspired systems.

**Figure 3:**
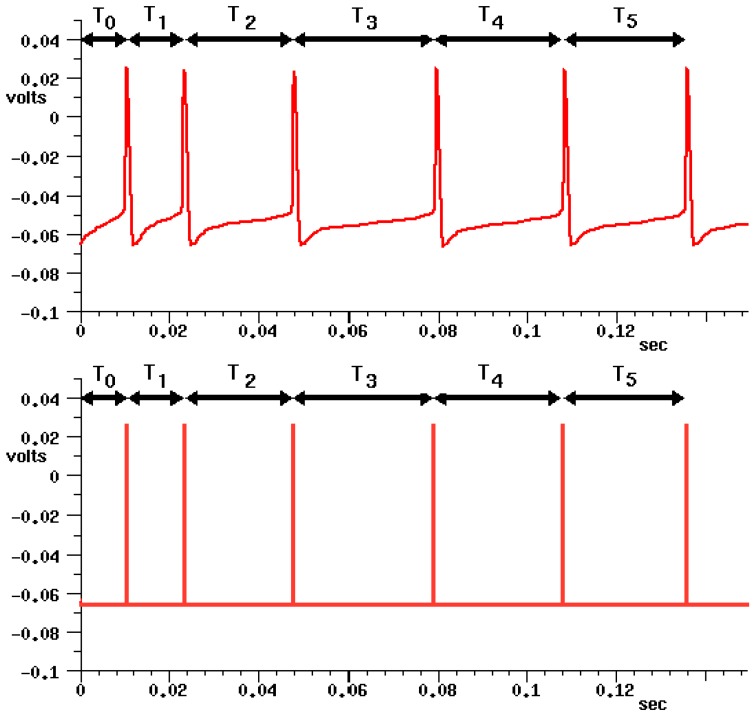
Representation of a neural pulse signal (above); and its electronic pulse equivalent (spike) PFM (below).

The action potentials, responsible for the communication between neurons, could be modeled as pulses with a constant pulse width and a variable frequency, as shown in Figure 3. Thus, PFM control is a modulation that shares some features with the action potentials and, therefore, it could be considered as a bioinspired control method.

In this paper, the design, manufacture and control of servomotors with PFM are presented.

The paper is organized as follows: In Section 2, we present the materials and methods used to build the controller where the microcontroller and the coding flow are shown. In Section 3, the results obtained are shown. Finally, the discussion in Section 4 includes a comparison with its peer PWM.

## 2. Materials and Methods

This section shows the details of the electronic components used and the steps taken to develop the design of the control circuit for the servomotor controller with PFM. The list of materials includes the servomotor, the driver and the microcontroller. The steps taken to design the controller include a prototype PCB, the control software (firmware) and the final PCB fabrication. Finally, to validate our controller, we have undertaken several studies on the new servomotor in terms of: power consumption, speed and boundaries. We will establish a performance comparison between PFM and PWM servomotors.

### 2.1. Servomotor

The servomotor used in this paper is 31311S from the brand Hitec (commonly known as Hitec HS-311 (the following website shows the technical specifications of the servomotor: https://servodatabase.com/servo/hitec/hs-311). The power supply for the motor ranges from 4.8 to 6.0 Volts and the speed range is correlated with the power supply ranging from 0.19/(60°) to 0.15/(60°).

### 2.2. Motor Driver

The Integrated Circuit (IC) chosen to drive the motor is the BD6210F-E2 of the brand ROHM Semiconductor. This IC can operate at a power supply voltage ranging from 3.0 V to 5.5 V, with output currents up to 2 A. The main features of this H-Bridge driver are: built-in one channel configuration, a pin called “VREF” which enables PWM duty control (although it is not of our interest), a cross-conduction prevention circuit and four extra protection circuits. Depending on the control signal, the driver moves the motor in one direction or the opposite direction.

### 2.3. Microcontroller

These are features we must address to select the microcontroller: the number of pins should be enough to allocate all the electronic components and the physical dimensions should not exceed the size of the motor case to allow its inclusion in the PCB. To meet these constraints, we have chosen the Atmel ATTiny84, an 8-bit microcontroller with 8 kBytes in-system programmable flash, 512 bytes of in-system programmable EEPROM and 512 bytes of SRAM. It works at 1 MHz by default, but it accepts external oscillators up to 20 MHz. The package selected is the 14S1 which, according to the manufacturer, results in a 6.19 × 8.74 mm^2^ dimension.

**Figure 4:**
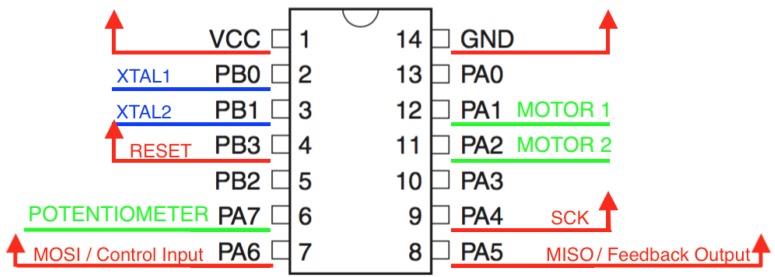
Connection diagram of the microcontroller ATTiny84. Internal connections (motor driver and potentiometer pinout) are marked in green. Cristal pinout is marked in blue. External pinouts for programming and power are marked in red. MOSI pin works as the control pin and MISO pin works as the feedback pin.

Figure 4 shows the pin out of the microcontroller that we have implemented. Since the microcontroller has the option to be programmed externally, we have routed these pins as output pins so the firmware of the micro could be updated at any time.

### 2.4. Firmware

We have developed a code for the microcontroller that processes the PFM signal received and makes the motor shaft spin in a particular direction. As we saw previously, pulse-width remains constant in a PFM signal, while frequency is what modulates the rotation angle of the shaft.

**Figure 5:**
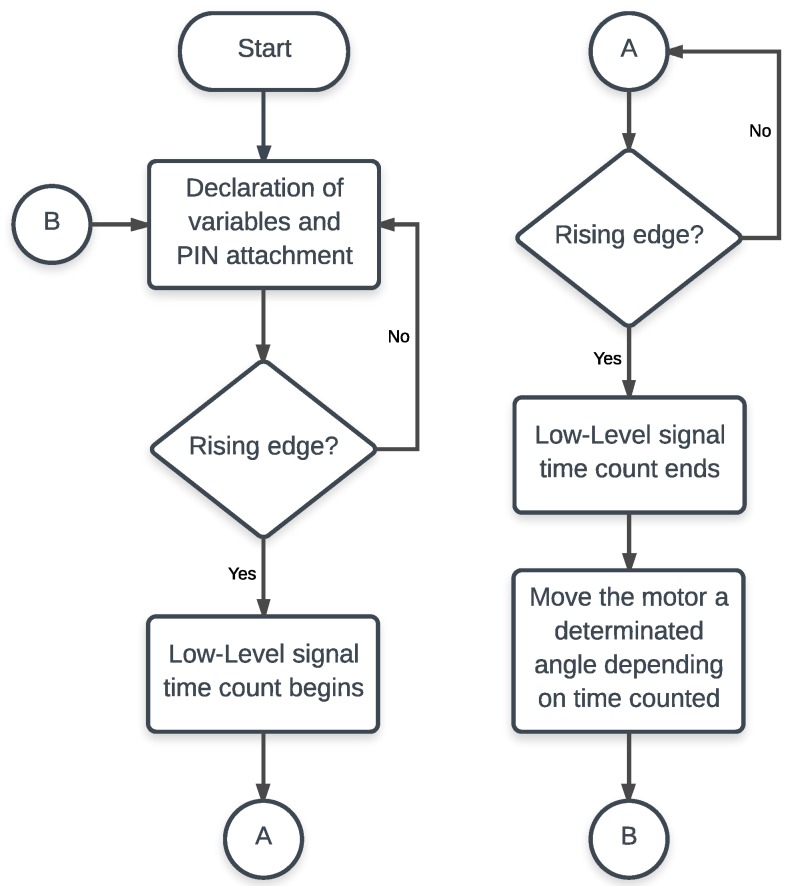
Flow chart of the firmware. The microcontroller sends an interruption for every rising edge detected in a declared pin. It starts counting the time when the signal is low-level, and compares this counter with the feedback of the potentiometer. In the case there is a difference, the motor is moved towards the set position.

The flow chart of the code is shown in Figure 5. A brief description: the period of the signal is measured detecting rising edges. The range of this value is predefined and it goes from 1 to 2 ms meaning a rotation angle between 180° and 0°. As a control system, this reference value is compared with the feedback given by the potentiometer and then, the microcontroller removes the control signal supplied to the driver whenever both values match.

The feedback signal is generated by the microcontroller. The output value of the potentiometer goes to the microcontroller where it is mapped to a value that ranges from 0° to 180°. Then, a PFM signal is generated according to that value. The frequency of the signal follows Equation (1).

(1) $$f_{PFM\_feedback}\;=\;2.78\;\times \;POT_{val}\;+500$$

The firmware could be updated any time after the motor is running using the device AVRISP MKII; a solution given by the Atmel Company to program its microcontrollers.

### 2.5. Control Technique

The controller will use both PFM signals (set-point and feedback), and it will generate an ON–OFF signal to feed the motor terminals through the driver. Thus, we use a bang-bang control technique.

## 3. Results

The controller proposed is introduced in the servomotor case by removing the original entire control circuitry connected to the DC motor and keeping all other parts included within the servomotor case. To provide feedback to the new controller, we used a 10 kΩ potentiometer as a sensor.

**Figure 6:**
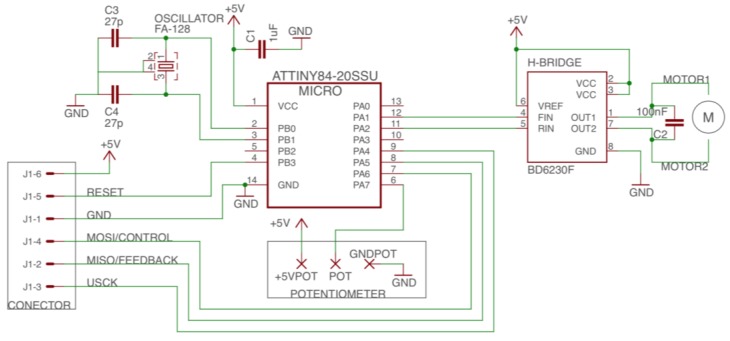
Schematic of the control circuit. J1 is the external connector that provides power supply and gives the flexibility of updating the microcontroller code. The potentiometer is connected to an analog pin of the microcontroller. An H-Bridge drives the motor in terms of current and a capacitor is added between the motor terminals for noise filtering purposes.

**Figure 7:**
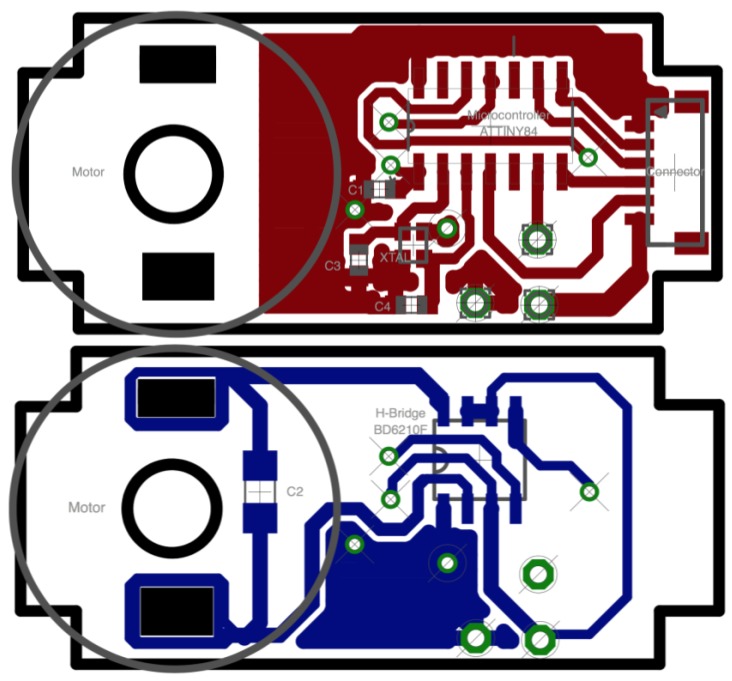
Control circuit board design: (**top**) the top layer of the PCB; and (**bottom**) the bottom layer.

**Figure 8:**
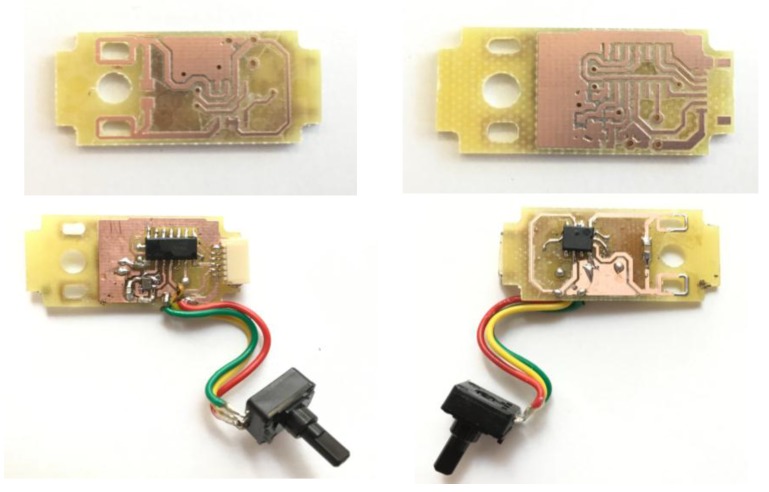
Prototype of the manufactured PCB. It has the precise size to fit inside the servomotor case.

The schematic of the controller design is shown in Figure 6, and the printed circuit board (PCB) is shown in Figure 7. The circuit includes the microcontroller and its interfaces with the output connector (J1), the driver, the 10 K potentiometer and the oscillator (an external oscillator of 16 MHz is added to allow higher frequencies and future improvements). The PCB designed fits inside the case of the servomotor (Figure 8).

The performance of the controller is measured with two different studies: first, an analysis of the behavior of a PFM controlled motor to check its boundaries and response; and, secondly, a comparison of two servomotors, one PFM controlled and one PWM controlled, in terms of speed and current consumption.

### 3.1. PFM Servomotor Analysis

The behavior of the servomotor controlled using PFM depends on two parameters: the duty-cycle (related to the pulse-width) of the signal sent to the motor driver and the frequency of this square signal.

The first test is made to check the behavior of the control circuit. There are two parameters to configure: the width of the pulse and the range of frequency for the PFM signal. The values of these parameters are selected according to the average values of the spike width [23] for the pulse width: 1 ms; and the average of firing rates [24] for the frequency: [0, 100] Hertz. This range is mapped within the 0°–180° angle range.

However, the same range observed in neurons for the firing rates cannot be used for our controller, since a zero frequency means that the microcontroller will not be able to detect either rising or falling edges. Thus, the maximum rate will be given by the minimum time to produce a PFM signal from the measurement of the potentiometer: 13 μs. Thus, we changed our ranges to [476.2, 990.1] Hz.

With this change, we get a pulse-with 20 times smaller than the first one, thus pulse frequency has been incremented.

Regarding pulse-width, it could be as small as possible, so we use values in the range of microseconds and we increase frequency by decreasing the function value range. The ATTiny84 microcontroller works at 16 MHz, so the frequency range could not be higher than this.

**Figure 9:**
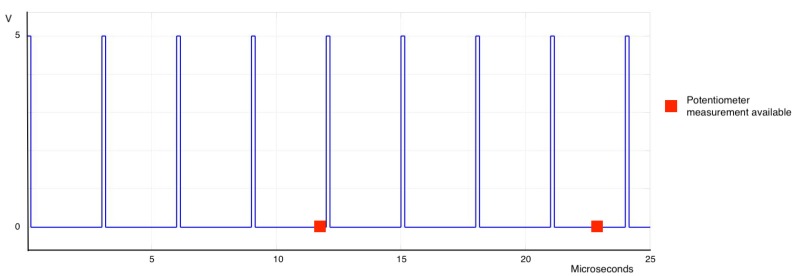
PFM signal with higher frequency than analog data acquisition. The red square shows whenever a new measurement from the potentiometer is available. That measurement will be the boundary for not losing information.

Nevertheless, the frequency of microcontroller is not the only value to consider. The analog data acquisition by the microcontroller is done every 13 μs. If the entire period of the signal is smaller than this value, the microcontroller could not detect every rising-edges of the signal, because the comparison of this value with the potentiometer happens every 13 μs. This results in data loss. An example is shown in the Figure 9.

**Figure 10:**
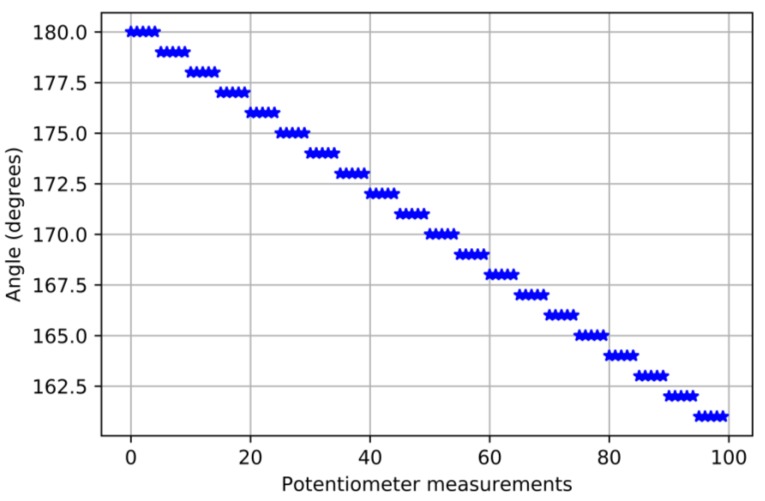
Mapping of the potentiometer values measured with the angle reached by the motor.

Since we use a 10-bits ADC, the value we measure from the potentiometer goes from 0 to 1023. This measurement is available every 13 μs. That means that we must map 1024 values into 180°. Thus, we have an over resolution on the potentiometer. Figure 10 shows how we have mapped the potentiometer measurements into degrees of the servomotor: five values in a row are mapped to 1°.

Thus, the final set-up for the parameters of the servomotor controller are: 1 μs pulse width, a frequency range of [476.2, 990.1] Hz and a tolerance of ±2.5° from the potentiometer measurements.

Finally, in this experiment and within this configuration, we observe an error between the reached and the set angle of 10°.

**Figure 11:**
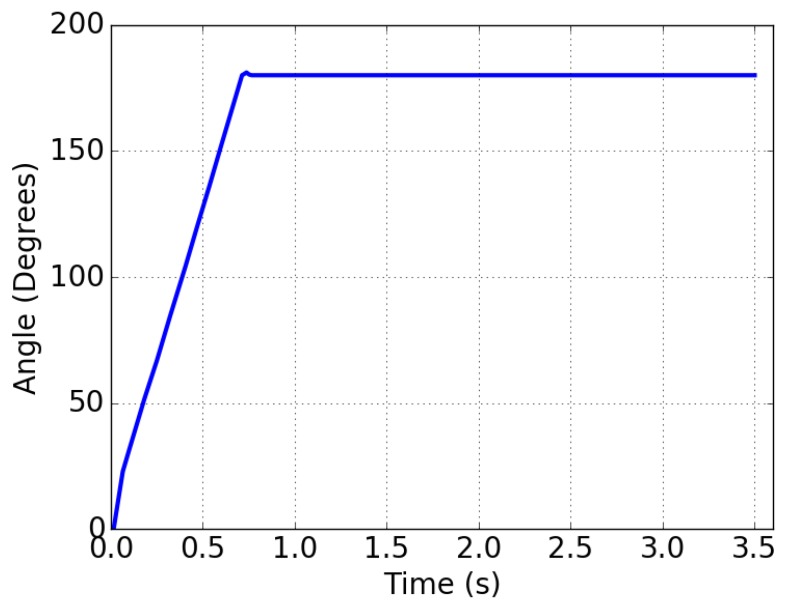
Response of the servomotor when the stimulus is changed from 0° to 180° at *t* = 0. It can be seen how there is an overshoot of 2° . The servomotor reached the set-point position.

Figure 11 shows the response of the final calibrated PFM servomotor when the set-pint is changed from 0° to 180° at *t* = 0 s.

### 3.2. Comparative Study between PFM and PWM Servomotors

When a servomotor is controlled using PWM, the squared signal sent to the motor has a fixed period of 20 ms, changing the pulse-width between 1 ms and 2 ms. Since, using our PFM controller, we can have a decision every 13 μs, it allows a faster modification of the set-point, reducing the latency of the control system.

**Figure 12:**
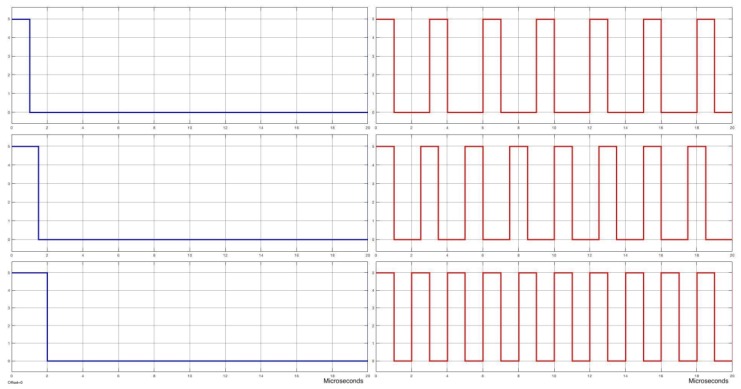
Pulse comparison between PWM and PFM signals at the same range time: (**Left**) PWM; and (**Right**) PFM.

Figure 12 shows a comparison when both techniques (PWM and PFM) are used. It can be seen that the amount of information is ten times higher when PFM is used. Moreover, pulse-width and frequency range could be updated to further increase this faster transference.

In terms of power consumption, the current consumption is shown in Table 1 for both modulations. Two measurements are made: the first when both servomotors are not driven at all, and the second when the servomotors command a movement from 0° to 180° (each set-point is supplied within 1 s).

The current consumption is higher when PFM is used since the added electronics are considered.

**Figure 13:**
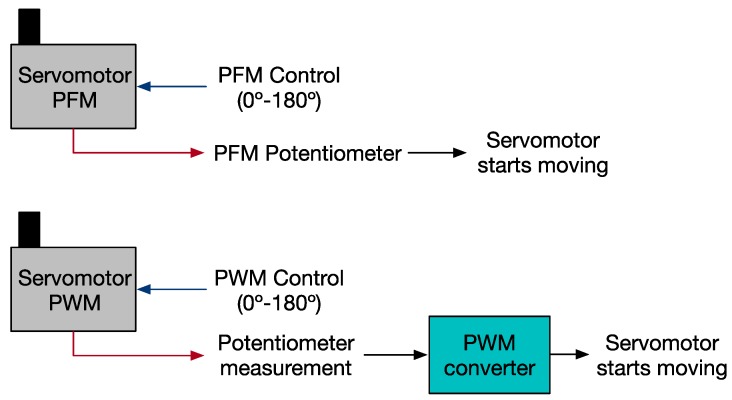
Block diagram about response speed study of servomotors. Both servomotors spin from 0° to 180°. Control and feedback signals are acquired for the following comparison and study.

Using the set-up shown in Figure 13, the performance of both a PWM and a PFM controlled servomotor is measured in terms of response speed. A logic analyzer was used to acquire both the PFM control signal and the PFM feedback signal generated from the potentiometer value. The potentiometer from the PWM controlled servomotor is converted, using an ADC, to the same frequency ranges of the PFM to allow a comparison between them. Then, the response speed is given by the difference of these two: a control signal is sent to the servo and the servo starts moving, i.e., the potentiometer is providing some feedback. 

As we can see, PFM servomotor is 700% faster at the start than the one driven by PWM.

The time to reach the set-point is also measured using the same setup as in Figure 13. The time is measured starting when the set-point is supplied and finishing when the servo reaches it (according to the feedback provided by the potentiometer).

The result shows that, in this case, the PFM modulation is slightly faster than the PWM one.

**Figure 14:**
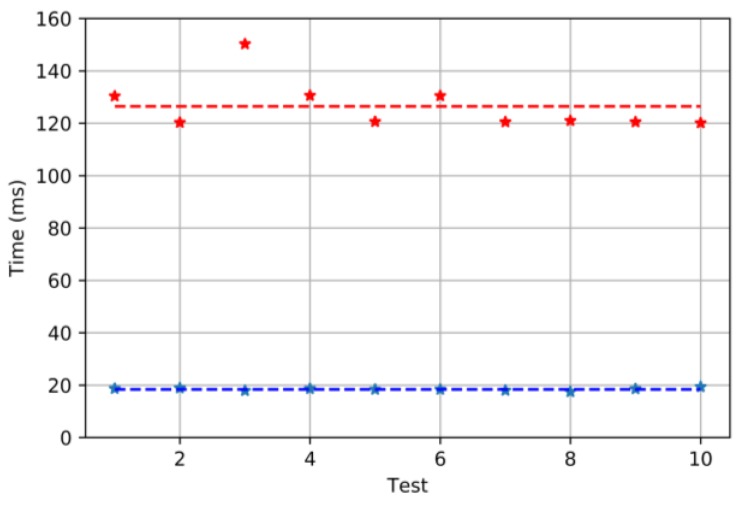
Ten tests run with both PFM and PWM controllers. The blue dots represent the angle reached during each test with PFM and the red dots when PWM is used. The average angle is also shown: 18.38 ms for the PFM and 126.44 for the PWM.

**Figure 15:**
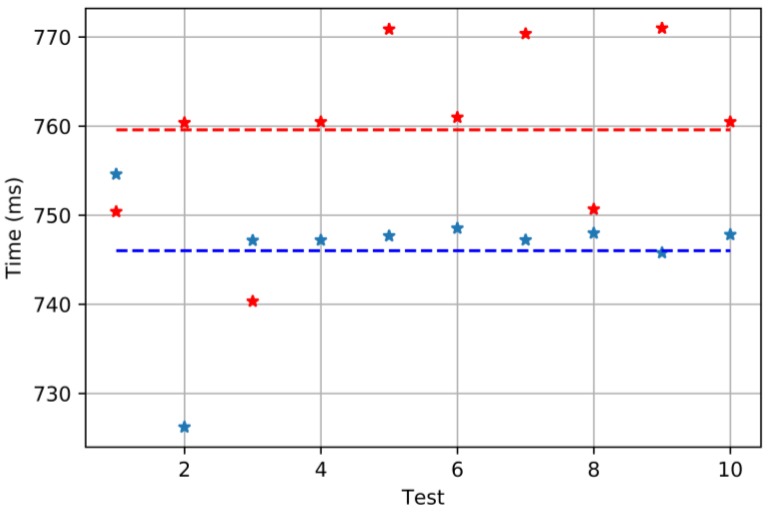
Steady state results when PFM and PWM are used. Ten tests are run for each modulation: in blue, the results for PFM, and, in red, for PWM. The average values are also shown: 746.01 ms for PFM and 759.58 ms for PWM. The set-point used was 180°.

As Figure 14 and Figure 15 show, our controller is faster than the PWM mode. Now, as a case study, a neuromorphic system is considered [12]; we designed a test to check if our controller improves the behavior. The test consists of sending a rate (neuron fashion-like) to the motor: one path is directly sent to the servo with our PFM controller and the other path includes a signal converter to adapt the pulse train to a PWM signal. Then, we measured the delay between both signals at the terminals of each servomotor.

**Figure 16:**
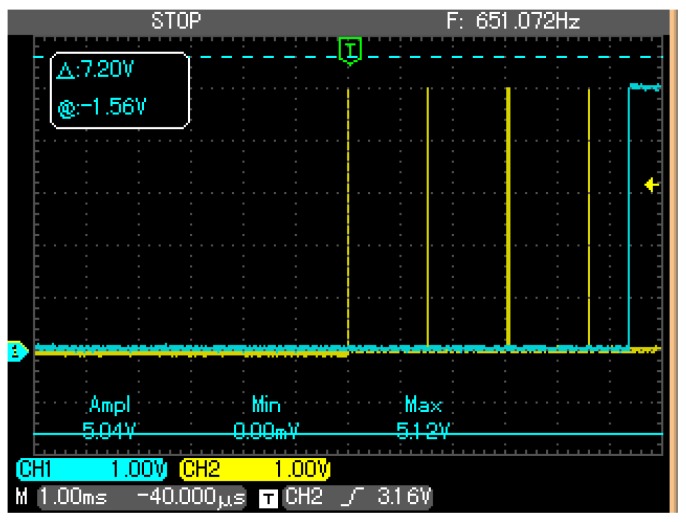
Oscilloscope screenshot to show the delay between the PFM signal (yellow) and the PWM one (blue).

Figure 16 shows the result: the PWM signal is delayed by 5360 μs.

Thus, with our device, in this case study, the delay introduced by the converter device is removed. Furthermore, the feedback signal from the potentiometer is externally available through a pin of the microcontroller.

## 4. Conclusions

The paper presented a control circuit to allow a commercial servomotor to be controlled using PFM. The PCB designed fits inside the motor case and a potentiometer is used as the sensor providing feedback information to the system. The microcontroller included can be externally interfaced to easily change the operation mode.

The controller presented is an improvement on the PWM version. The frequency of a PWM signal is usually 50 Hz. Our design samples the potentiometer every 13 μs; thus, we could have a new error signal computed shortly after, which means that our PFM controller can reach higher frequencies than the PWM.

The results show a higher transmission of information in the same time range for PFM signals than PWM ones. This higher speed of pulses, plus the stability, make PFM controlled servomotors appropriate devices to be used with bioinspired systems, as was suggested in [25].

This controller represents an ideal way of allowing roboticists or neuromorphic engineers to use the servomotors directly interfaced with the output signal of a neural network, thus removing the delay previously introduced by the conversion to PWM.

To our knowledge, there are no previous works similar to ours. This demonstrates the originality of this paper.

## Figures and Tables

**Table 1:** Current consumption of the PWM and PFM servomotors.

	PFM Servomotor	PWM Servomotor
Stopped	12.6 mA	6.9 mA
0°–180°	140–176.5 mA	84–135 mA
Delay: 1 s		
